# Chemerin induces endothelial cell inflammation: activation of nuclear factor-kappa beta and monocyte-endothelial adhesion

**DOI:** 10.18632/oncotarget.24659

**Published:** 2018-03-30

**Authors:** Georgios K. Dimitriadis, Jaspreet Kaur, Raghu Adya, Alexander D. Miras, Harman S. Mattu, John G. Hattersley, Gregory Kaltsas, Bee K. Tan, Harpal S. Randeva

**Affiliations:** ^1^ Division of Translational and Experimental Medicine, Warwick Medical School, University of Warwick, Coventry, UK; ^2^ Division of Endocrinology and Experimental Medicine, Imperial College London, Hammersmith Campus, London, UK; ^3^ WISDEM Centre, Human Metabolism Research Unit, University Hospitals Coventry and Warwickshire NHS Trust, Coventry, UK; ^4^ Division of Biomedical Sciences, School of Medicine, University of California, Riverside, CA, USA; ^5^ Department of Obstetrics and Gynaecology, Birmingham Heartlands Hospital, Heart of England NHS Foundation Trust, Birmingham, UK; ^6^ Division of Life and Health Sciences, Aston University, Birmingham, UK

**Keywords:** chemerin, T2DM, atherosclerosis, endothelium, inflammation

## Abstract

Chemerin, a chemoattractant protein, acts *via* a G-protein coupled chemokine receptor, *i.e.* Chemokine like Receptor 1/ChemR23; levels of which are elevated in pro-inflammatory states such as obesity and type 2 diabetes mellitus (T2DM). Obesity and T2DM patients are at high risk of developing cardiovascular disorders such as atherosclerosis. We have reported that chemerin induces human endothelial cell angiogenesis and since dysregulated angiogenesis and endothelial dysfunction are hallmarks of vascular disease; we sought to determine the effects of chemerin on monocyte-endothelial adhesion, and nuclear factor kappa-light-chain-enhancer of activated B cells (NF-κB), a critical pro-inflammatory transcription factor. Human endothelial cells were transfected with pNF-kappaB-Luc plasmid. Chemerin induced NF-κB activation *via* the MAPK and PI3K/Akt pathways. Western blot analyses and monocyte-endothelial adhesion assay showed that chemerin increased endothelial cell adhesion molecule expression and secretion, namely E-selectin (Endothelial Selectin), VCAM-1 (Vascular Cell Adhesion Molecule-1) and ICAM-1 (Intracellular Adhesion Molecule-1), leading to enhancement of monocyte-endothelial adhesion. Additionally, we showed a synergistic response of the pro-inflammatory mediator, Interleukin-1β with chemerin induced effects. Chemerin plays an important role in endothelial inflammation, as it induces monocyte-endothelial adhesion, a critical step in the development of atherosclerosis.

## INTRODUCTION

Obesity, characterised as a state of chronic low grade systemic inflammation, is recognised as an independent risk factor for cardiovascular morbidity and mortality and a significant contributor to the pathogenesis of atherosclerosis [1]. Interactions between endothelial cells and circulating monocytes, in co-existing arterial wall dysfunction and systemic inflammation, are hallmarks of this complex pathophysiological process [2]. Various adipose tissue derived bio-active molecules (adipokines) have been implicated in the maintenance of vascular homeostasis including expression and engagement of adhesion molecule receptors on both endothelial cells and monocytes such as E-selectin (endothelial selectin), vascular cell adhesion molecule 1 (VCAM-1) and intercellular adhesion molecule-1 (ICAM-1) [3]. Altered circulating adipokine concentrations have been reported in obese, insulin resistant subjects with T2DM [4–7]. Activation of these endothelial adhesion molecules *via* the NF-кB pathway is an important step in vascular inflammation and the development of atherosclerosis [8].

A recently discovered adipokine, chemerin, functions as a chemo-attractant protein, mediating its effects through a G-protein Coupled Receptor, Chemokine like Receptor 1, also known as ChemR23 [9]. Increased circulating levels of chemerin are found in obesity, exhibiting positive correlations with various aspects of the metabolic syndrome [10]. Studies have implicated the role of chemerin and Chemokine like Receptor 1 in the recruitment of immune cells in inflammatory and auto-immune disorders [11]. More importantly Spiroglou *et al*. have demonstrated increased periaortic and pericoronary adipose tissue expression levels of chemerin in patients with atherosclerosis, suggesting a possible paracrine mediated effect [12]. Additionally, we have previously demonstrated the pro-angiogenic effects of chemerin [13]. Dysregulated angiogenesis, in particular plaque neovascularisation, is a recognised contributor to symptomatic atherosclerosis [14]. NF-B is a major transcription factor in inflammatory responses, regulating a plethora of genes, pivotal in the initiation and progression of vascular inflammation [15]. Therefore, we investigated the effects of chemerin on monocyte-endothelial interaction, the involvement of endothelial specific adhesion molecules and the participation of NF-кB, MAPK and PI3Akt pathways.

## RESULTS

### Chemerin activates NF-кB in HMEC-1 cells *via* MAPK and PI3K/Akt pathways; synergistic activation with IL-1β

**Figure 1:**
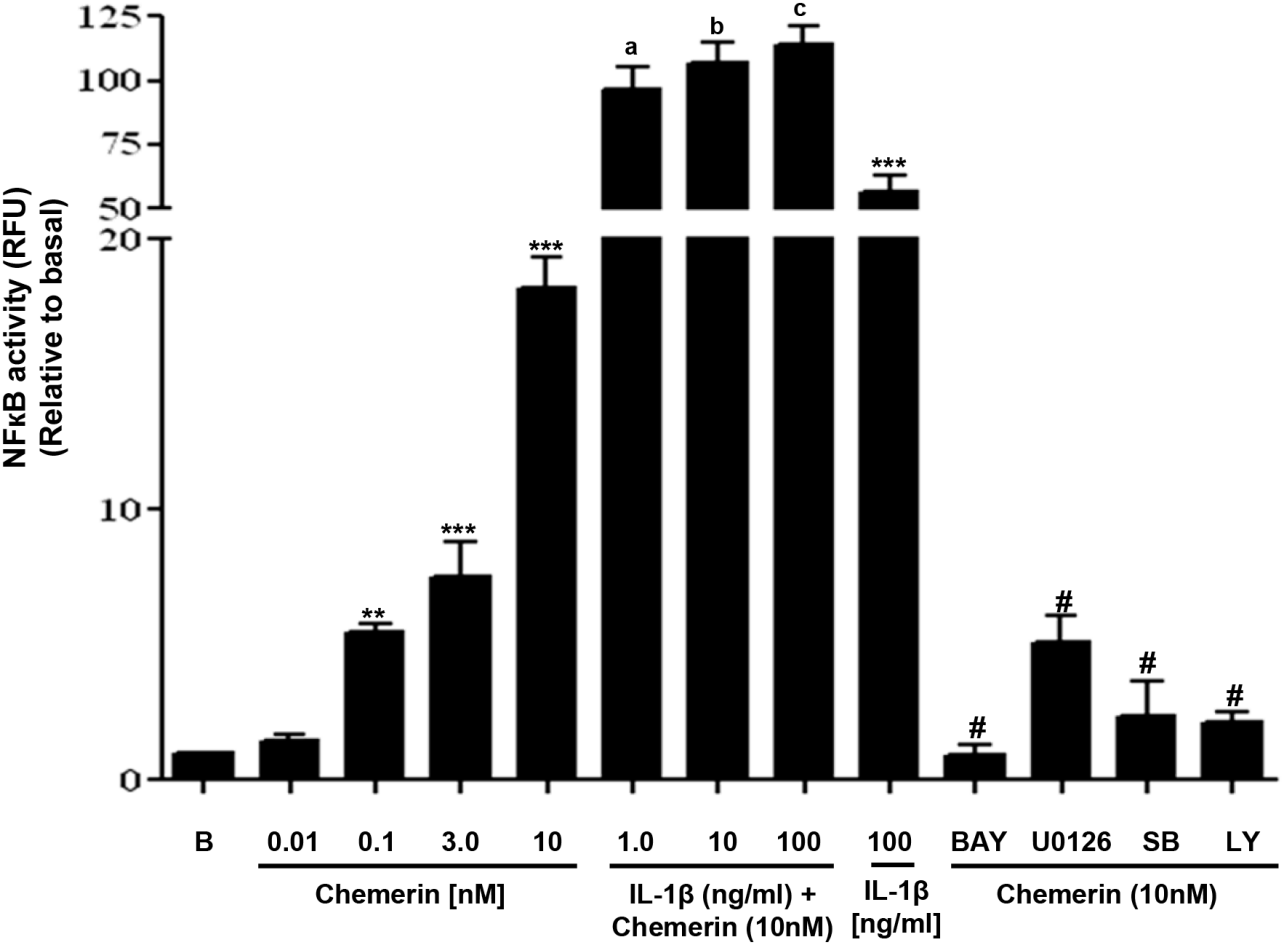
Chemerin activates NF-кB in HMEC-1 cells *via* MAPK and PI3K/Akt pathways; synergistic activation with IL-1β pathways Serum starved HMEC-1 cells stably transfected with pNFкB-Luciferase were treated with or without chemerin (0-10nM) for 2 hours. Cells were lysed and luciferase activities were measured. Chemerin induced a concentration dependent increase in luciferase activity after 2 hours of incubation (^**^P < 0.01 vs. basal, ^***^P < 0.001 vs. basal). When pre-incubated with BAY 11-7085 [(10μM), NF-ĸB inhibitor] or U0126 [(10μM), MAPK inhibitor] or SB202190 [(1 μM), p38 MAPK inhibitor] or LY294002 [(10 μM), PI3K/Akt inhibitor] for 1 hour, chemerin induced (10nM) NF-B activation was significantly attenuated (^#^*P* < 0.001 vs. chemerin (10nM) only treated). When chemerin (10nM) was co-incubated with IL-1β (0-100ng/mL), a significant increase in NF-B activity was observed [Figure 1: ^a^*P* < 0.05, ^b^*P* < 0.01, ^c^*P* < 0.001 vs. IL-1β (100ng/mL) only treated, *P* < 0.001 vs. chemerin (10nM) only treated]. Data are mean ± SE of three experiments. Each experiment was carried out in three replicates. Group comparison by ANOVA (post hoc analysis: Tukey's test).

We employed a NF-кB-Luc plasmid, stably transfected Human Microvascular Endothelial Cell (HMEC)-1 cell line to investigate the role of NF-кB in the development of vascular inflammation. Our findings were that chemerin treatment increased the luciferase activity in a concentration-dependent manner after 2 hours of incubation (Figure 1: ^**^P < 0.01 vs. basal, ^***^P < 0.001 vs. basal). However, when pre-incubated with BAY 11-7085 [(10μM), NF-ĸB inhibitor] or U0126 [(10μM), MAPK inhibitor] or SB202190 [(1 μM), p38 MAPK inhibitor] or LY294002 [(10 μM), PI3K/Akt inhibitor] for 1 hour, chemerin induced (10nM) NF-B activation was significantly attenuated [Figure 1: ^#^*P* < 0.001 vs. chemerin (10nM) only treated]. Furthermore, when chemerin (10nM) was co-incubated with IL-1β (0-100ng/mL), a significant increase in NF-B activity was observed [Figure 1: ^a^*P* < 0.05, ^b^*P* < 0.01, ^c^*P* < 0.001 vs. IL-1β (100ng/mL) only treated, *P* < 0.001 vs. chemerin (10nM) only treated].

### Chemerin increases endothelial cell adhesion molecules mRNA, protein expression and secretion in HMEC-1 cells

**Figure 2:**
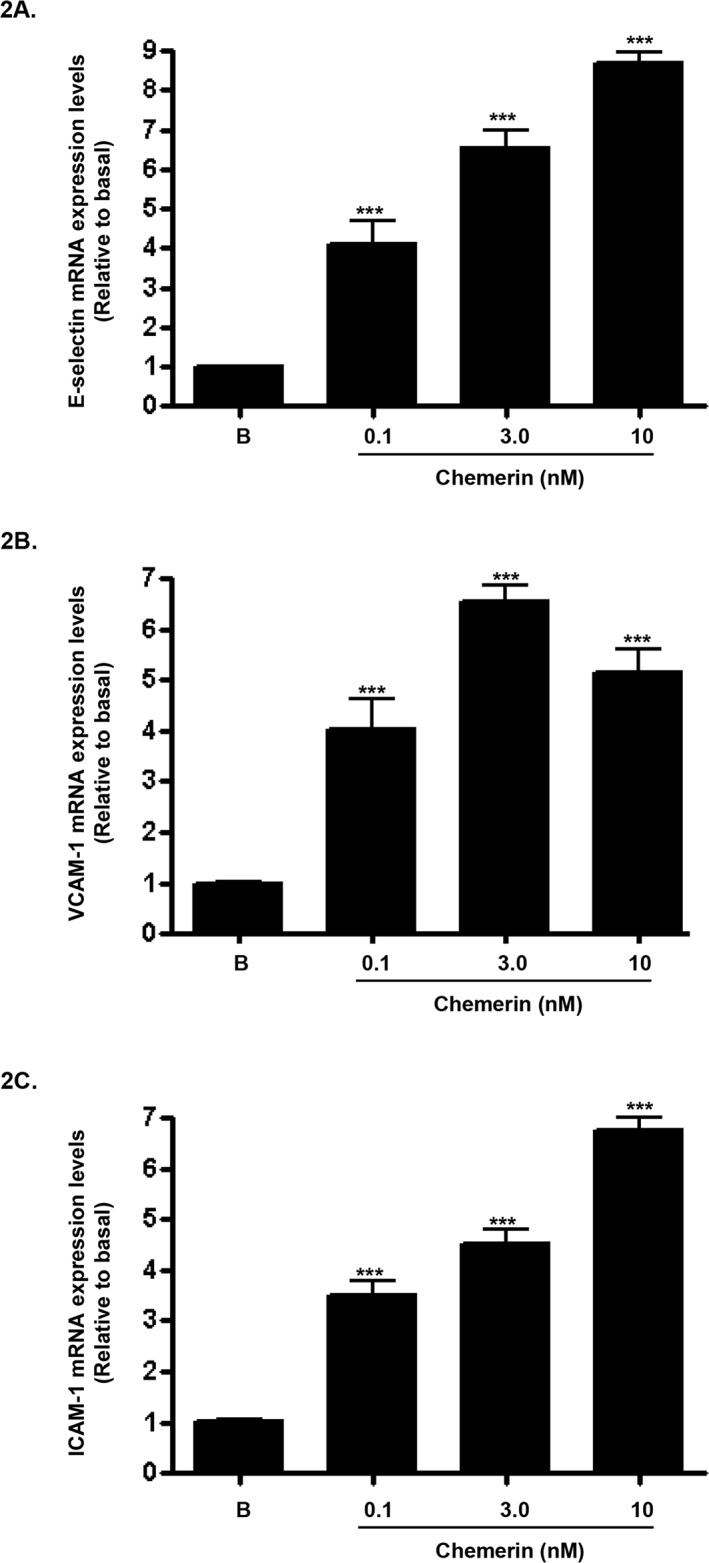
Chemerin increases endothelial cell adhesion molecules mRNA expression in HMEC-1 cells Serum starved HMEC-1 cells were treated with chemerin (0-10nM) for 4 hours. Real-time quantitative RT-PCR analyses showed that mRNA expression of cell adhesion molecules, *i.e.* E-selectin, VCAM-1 and ICAM-1, were significantly up-regulated by chemerin in a concentration dependent manner at 4 hours (Figures **2A-2C**: ^***^P < 0.001 vs. basal). Data are mean ± SE of three experiments. Each experiment was carried out in three replicates. Group comparison by ANOVA (post hoc analysis: Tukey's test).

**Figure 3:**
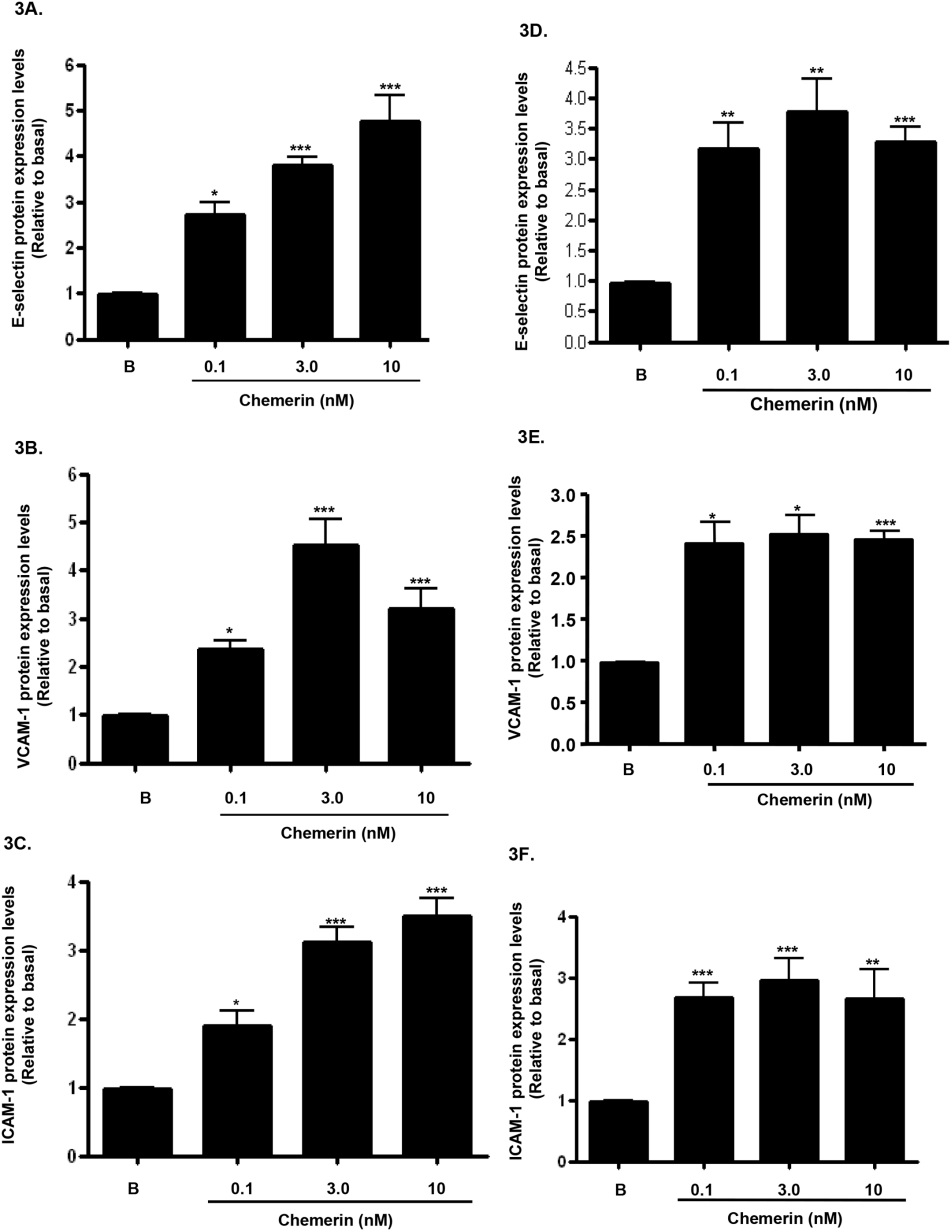
Chemerin increases endothelial cell adhesion molecules protein expression in HMEC-1 cells Serum starved HMEC-1 cells were treated with chemerin (0-10nM) for 12 and 24 hours. Densitometric analysis of western blots (cell protein lysates) of E-selectin, VCAM-1 and ICAM-1 immune complexes having normalized to β-actin, respectively, showed that protein expression of cell adhesion molecules, *i.e.* E-selectin, VCAM-1 and ICAM-1, were significantly increased by chemerin in a concentration dependent manner at 12 hours (Figures **3A-3C**: ^*^P < 0.05 vs. basal, ^***^P < 0.001 vs. basal) and 24 hours (Figures **3D-3F**: ^*^P < 0.05 vs. basal, ^**^P < 0.01 vs. basal, ^***^P < 0.001 vs. basal). Data are mean ± SE of three experiments. Each experiment was carried out in three replicates. Group comparison by ANOVA (post hoc analysis: Tukey's test).

**Figure 4:**
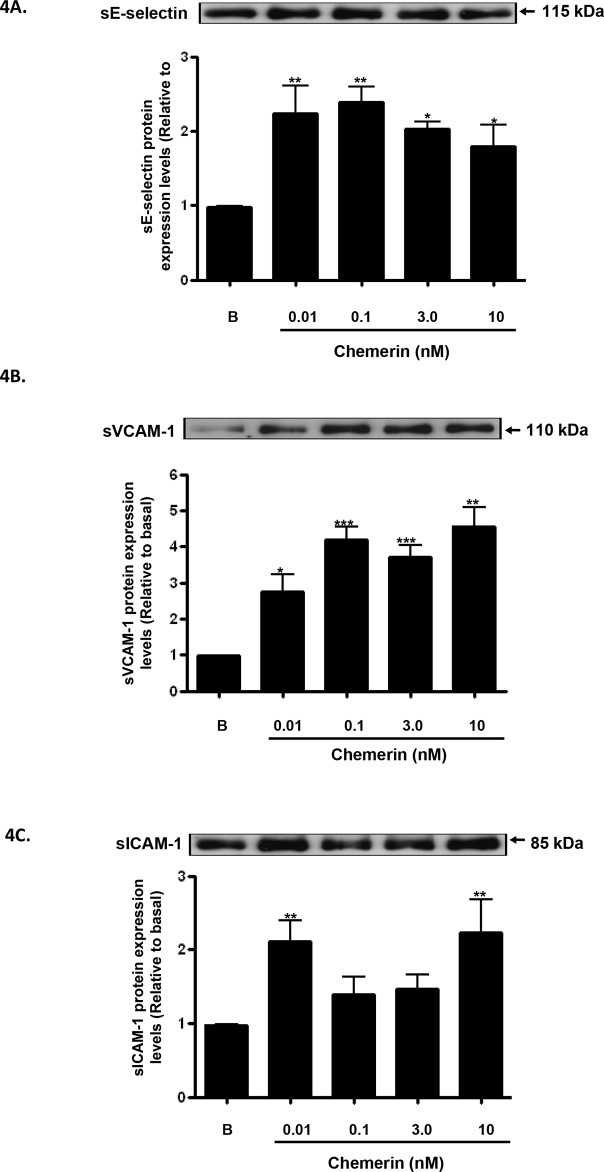
Chemerin increases endothelial cell adhesion molecules protein secretion in HMEC-1 cells Serum starved HMEC-1 cells were treated with chemerin (0-10nM) for 12 hours. Densitometric analysis of western blots (conditioned media) of E-selectin, VCAM-1 and ICAM-1 immune complexes having normalized to β-actin, respectively, showed that secretion of cell adhesion molecules, *i.e.* E-selectin, VCAM-1 and ICAM-1, were significantly elevated by chemerin in a concentration dependent manner at 12 hours (Figures **4A-4C**: ^*^P < 0.05 vs. basal, ^**^P < 0.01 vs. basal, ^***^P < 0.001 vs. basal). Data are mean ± SE of three experiments. Each experiment was carried out in three replicates. Group comparison by ANOVA (post hoc analysis: Tukey's test).

Increased expression of endothelial cell adhesion molecules are hallmarks of vascular inflammation and atherosclerosis [19]. Serum starved HMEC-1 cells were stimulated with chemerin (R&D; 0-10nM) for 4, 12 and 24 hours, following initial concentration and time dependent optimisation experiments (data not shown). Real-time quantitative RT-PCR analyses showed that mRNA expression of E-selectin, VCAM-1 and ICAM-1, were significantly up-regulated by chemerin in a concentration dependent manner at 4 hours (Figures 2A-2C: ^***^P < 0.001 vs. basal). Also, western blotting analyses of HMEC-1 cell protein lysates showed that protein expression of E-selectin, VCAM-1 and ICAM-1, were significantly increased in a concentration dependent manner at 12 hours (Figures 3A-3C: ^*^P < 0.05 vs. basal, ^***^P < 0.001 vs. basal) and 24 hours (Figures 3D-3F: ^*^P < 0.05 vs. basal, ^**^P < 0.01 vs. basal, ^***^P < 0.001 vs. basal). Furthermore, western blotting analyses of HMEC-1 cell conditioned media showed that secretion of E-selectin, VCAM-1 and ICAM-1, were significantly elevated by chemerin in a concentration dependent manner at 12 hours (Figures 4A-4C: ^*^P < 0.05 vs. basal, ^**^P < 0.01 vs. basal, ^***^P < 0.001 vs. basal) and 24 hours (data not shown).

### Chemerin and IL-1β co-incubation; synergistic increase in cell adhesion molecule expression

**Figure 5:**
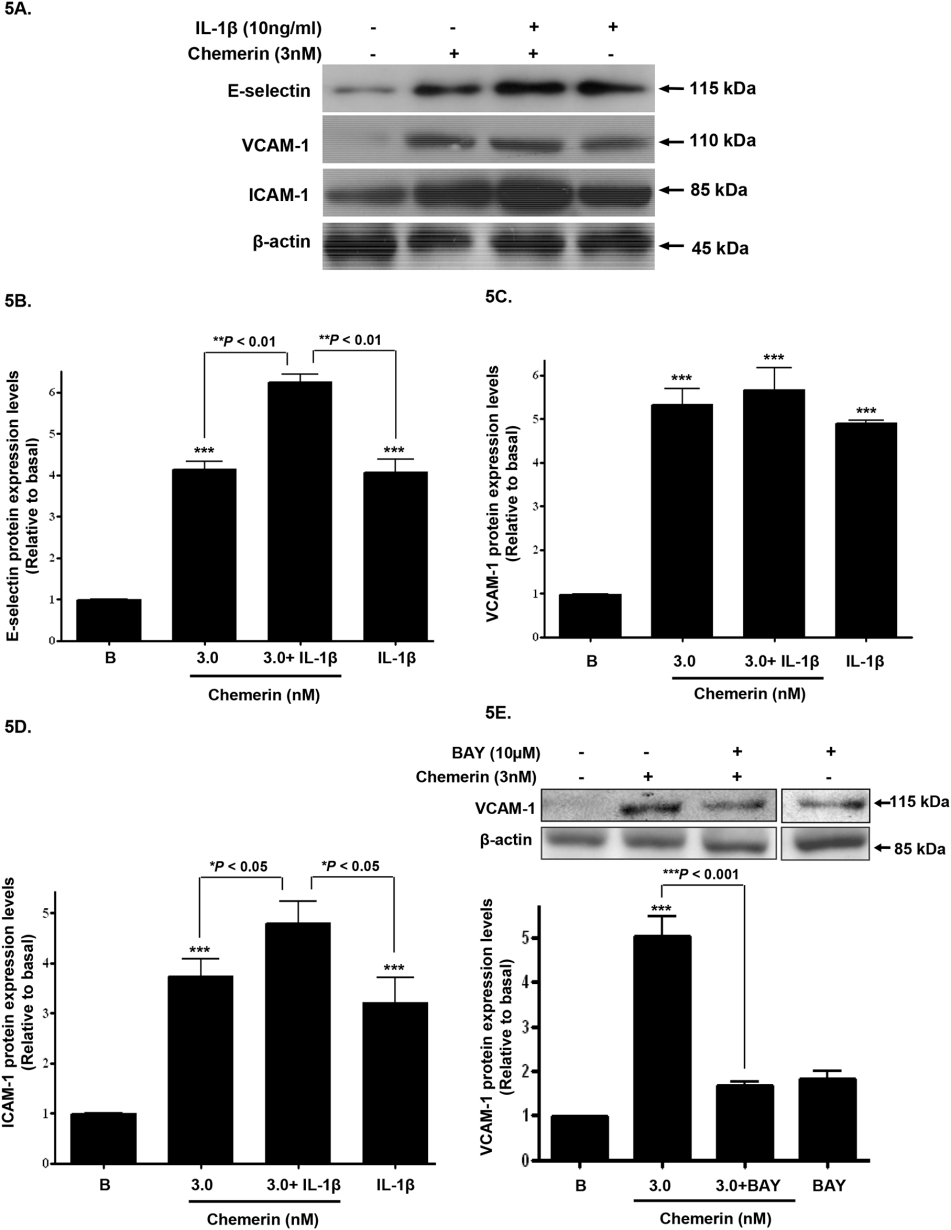
Chemerin and IL-1β co-incubation; synergistic increase in cell adhesion molecule expression Serum starved HMEC-1 cells were treated with chemerin (3nM) with or without IL-1β (10ng/ml) for 12 hours. (**A**) Representative western blots of E-selectin, VCAM-1 and ICAM-1 and their respective β-actin. (**B-D**) Densitometric analysis of western blots (cell protein lysates) of E-selectin, VCAM-1 and ICAM-1 immune complexes having normalized to β-actin, respectively, showed that protein expression of cell adhesion molecules, *i.e.* E-selectin, VCAM-1 and ICAM-1, were synergistically increased when compared to chemerin or IL-1β, alone (Figures 5A-5D: ^***^P < 0.001 vs. basal, ^*^P < 0.05 vs. chemerin or IL-1β alone, ^**^P < 0.01 vs. chemerin or IL-1β alone). (**E**) When pre-incubated with BAY 11-7085 [(10μM), NF-ĸB inhibitor], chemerin (3nM) induced VCAM-1 protein expression was significantly attenuated (Figure 5E: ^***^P < 0.001). Data are mean ± SE of three experiments. Each experiment was carried out in three replicates. Group comparison by ANOVA (post hoc analysis: Tukey's test).

Serum starved HMEC-1 cells were stimulated with chemerin (3nM) with or without IL-1β (10ng/ml) for 12 hours, following initial concentration and time dependent optimisation experiments (data not shown); western blotting analyses of HMEC-1 cell protein lysates showed that protein expression of E-selectin, VCAM-1 and ICAM-1, were synergistically increased when compared to chemerin or IL-1β alone (Figures 5A-5D: ^***^P < 0.001 vs. basal, ^*^P < 0.05 vs. chemerin or IL-1β alone, ^**^P < 0.01 vs. chemerin or IL-1β alone). In addition, when pre-incubated with BAY 11-7085 [(10μM), NF-ĸB inhibitor], chemerin (3nM) induced VCAM-1 protein expression was significantly attenuated, indicating the potential role of NF-ĸB in chemerin induced cell adhesion molecule expression (Figure 5E: ^***^P < 0.001). Furthermore, densitometric analysis of western blots from starved HMEC-1 treated with chemerin (0-10nM) cells, revealed significantly elevated secretion of E-selectin, VCAM-1 and ICAM-1, in a concentration dependent manner at 12 hours (Figures 4A-4C: ^*^P < 0.05 vs. basal, ^**^P < 0.01 vs. basal, ^***^P < 0.001 vs. basal).

### Chemerin stimulates monocyte-endothelial cell adhesion *via* NF-ĸB, MAPK and PI3K/Akt pathways

**Figure 6:**
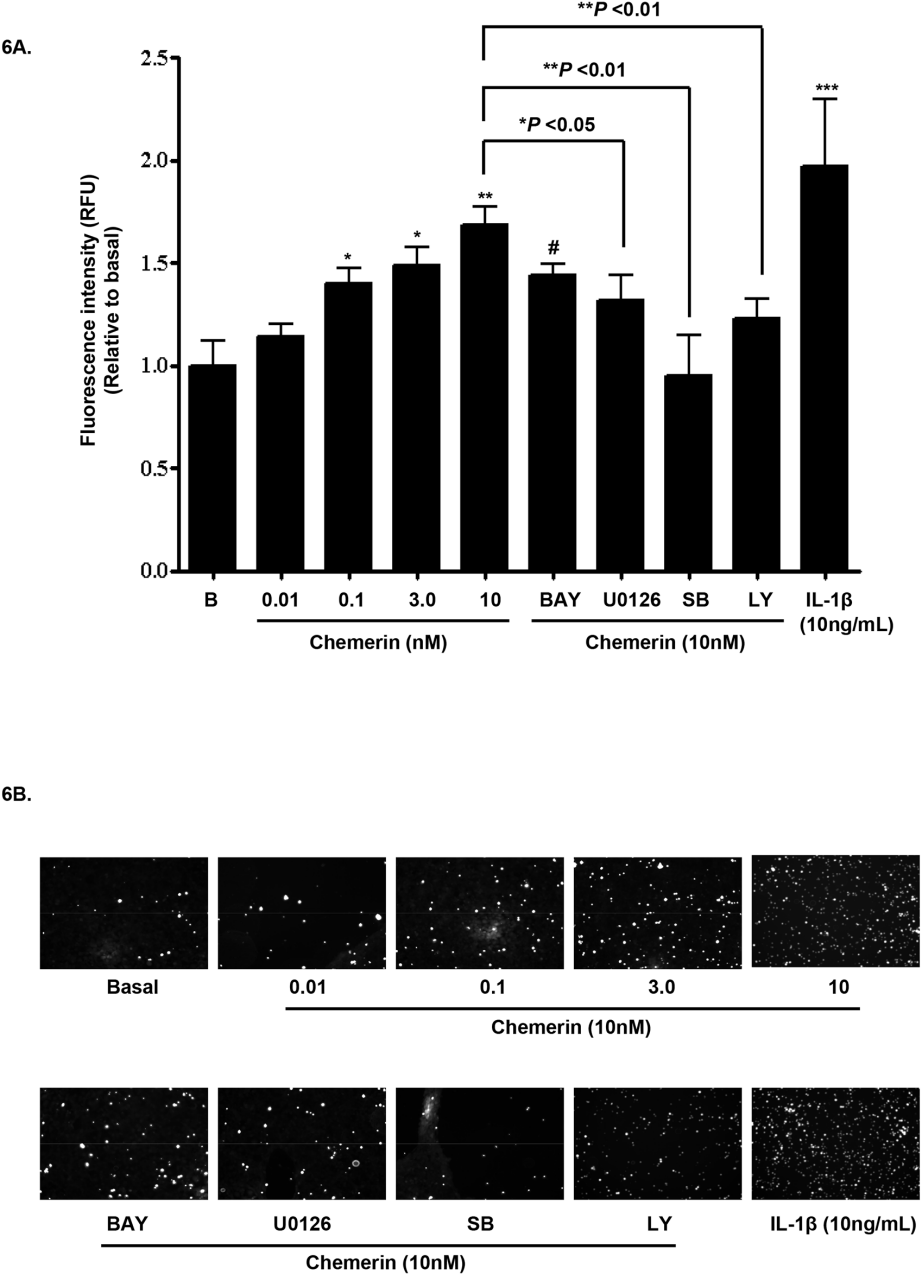
Chemerin stimulates monocyte-endothelial cell adhesion *via* NF-ĸB, MAPK and PI3K/Akt pathways Serum starved chemerin (0-10nM) treated HMEC-1 cells were co-cultured with THP-1 cells for 2 hours. Chemerin enhanced monocyte-endothelial adhesion in a concentration dependent fashion (Figure **6A**: ^*^P < 0.05 vs. basal, ^**^P < 0.01 vs. basal). Furthermore, pre-incubation for 1 hour with BAY 11-7085 [(10μM), NF-ĸB inhibitor] or U0126 [(10μM), MAPK inhibitor] or SB202190 [(1 μM), p38 MAPK inhibitor] or LY294002 [(10 μM), PI3K/Akt inhibitor], prior to treatment with chemerin (10nM) for 2 hours, chemerin induced monocyte-endothelial adhesion was significantly negated [Figure 6A: ^*^P < 0.05 vs. chemerin (10nM) only treated, ^**^P < 0.01 vs. chemerin (10nM) only treated]. The number of adherent cells is directly proportional to the fluorescence detected by a fluorescence plate reader expressed as Relative Fluorescence Units (RFU). Figure **6B** are representative fluorescent microscopy images, which were transferred using National Institutes of Health (USA) ImageJ software. Data are mean ± SE of three experiments. Each experiment was carried out in three replicates. Group comparison by ANOVA (post hoc analysis: Tukey's test).

Given that monocyte-endothelial interactions play a pivotal role in endothelial inflammation and the development of atherosclerosis [2], we assessed the effects of chemerin on monocyte-endothelial adhesion *in vitro*. Serum starved chemerin (0-10nM) treated HMEC-1 cells were co-cultured with Human Monocytic Leukemia Cells (THP-1) for 2 hours. Chemerin enhanced monocyte-endothelial adhesion in a concentration dependent fashion (Figure 6A: ^*^P < 0.05 vs. basal, ^**^P < 0.01 vs. basal). Furthermore, pre-incubation for 1 hour with BAY 11-7085 [(10μM), NF-ĸB inhibitor] or U0126 [(10μM), MAPK inhibitor] or SB202190 [(1 μM), p38 MAPK inhibitor] or LY294002 [(10 μM), PI3K/Akt inhibitor], prior to treatment with chemerin (10nM) for 2 hours, chemerin induced monocyte-endothelial adhesion was significantly ameliorated [Figure 6A: ^*^P < 0.05 vs. chemerin (10nM) only treated, ^**^P < 0.01 vs. chemerin (10nM) only treated], thereby implicating the role of NF-ĸB, MAPK and PI3K/Akt pathways in chemerin induced monocyte-endothelial adhesion. These results were further confirmed by fluorescent microscopy and images were transferred using National Institutes of Health (USA) ImageJ software (Figure 6B).

## DISCUSSION

Studies have supported an important role for chemerin in the pathophysiological processes involved in obesity and diabetes [10–13]. Clinical reports show elevated circulating levels of chemerin in these states with positive correlations to various aspects of the metabolic syndrome. We have previously demonstrated significantly increased serum chemerin levels in women with polycystic ovary syndrome (PCOS) [20]; a dysmetabolic state of pro-inflammation in women [21], associated with an increased risk of developing T2DM and cardiovascular disorders [22]. Furthermore, chemerin tissue expression levels are increased in periaortic and epicardial adipose tissue depots in subjects with atherosclerosis, implicating a paracrine mediated action of chemerin [12]. Similarly, Neves et al. demonstrated that chemerin plays an important role in molecular and cellular processes associated with vascular injury and dysfunction through vascular smooth muscle cells (VSMC) oxidative stress and inflammation in a ChemR23-inhibitable manner [23, 24]. Since endothelial cells are the first line of contact with circulating mediators of inflammation, it remains an important therapeutic target in both prevention and inhibiting the progression of cardiovascular disorders. In the present study, we report that chemerin activates a pivotal pro-inflammatory transcriptional regulator NF-кB in human endothelial cells. We also demonstrate that chemerin enhances expression and secretion of endothelial cell adhesion molecules (E-selectin, VCAM-1 and ICAM-1), and promotes monocyte-endothelial adhesion.

In vascular inflammatory responses, NF-κB signaling is an important regulator of endothelial cell adhesion molecules and initiator of atherosclerotic changes including vessel wall remodeling, as part of an inflammatory response [8]. In our experiments, in addition to chemerin induced NF-κB activation, a synergistic response was observed when chemerin was co-incubated with IL-1β. *In vivo* studies have demonstrated that IL-1β induced atherogenic action by enhancing expression of cell adhesion molecules, leading to recruitment of monocytes [24, 25]. This is very likely the reason for the enhanced effect observed when chemerin was co-incubated with IL-1β and similar findings have been reported by others using other cytokines; though the exact aetiology for this remains elusive. Cheshire *et al*. demonstrated the importance of cytokine induced synergistic activation of NF-кB in pro-inflammatory states resulting in potent activation of NF-кB-dependent gene expression including cell adhesion molecules [21]. Taken together with our findings, it is plausible to suggest that chemerin may be involved in the enhancement of endothelial activation in combination with other cytokines in a pro-inflammatory milieu.

In contrast, Provoost *et al.* demonstrated an opposing role for chemR23 signaling of inflammatory responses in the lungs, comparing chemR23 knockout (KO) mice with corresponding wild-type (WT) mice, depending on the context of traffic-related particulate matter. The chemR23 axis showed proinflammatory properties in a model of acute lung inflammation, in contrast to anti-inflammatory effects in a model of allergic airway inflammation [26]. Similarly, Laranjeira et al. also report anti-inflammatory properties for chemerin [27]. The underlying basis of these opposing activities of chemerin/chemR23 signaling remains unclear. Various proteases can process prochemerin to bioactive chemerin. Thus, depending on the proteases that are present in the microenvironment/organ, diverse chemerin fragments may be produced with distinct pharmacological properties [26].

Of late, it is thought that deciphering NF-κB linked multiple signaling cross talk is critical in the identification of novel therapeutic targets in combating atherosclerosis and related pathologies. Activation of NF-κB signaling involves and influences multiple signaling cascades including MAPK and PI3K/Akt pathways [28–31]. Our experiments demonstrated the involvement of MAPK and PI3K/Akt pathways in chemerin induced NF-κB activation. However, further research is needed to elucidate the interactions between these complex regulatory pathways.

NF-κB activation leads to transcription activation of gene promoter regions of cell adhesion molecules namely E-selectin, VCAM-1 and ICAM-1 [8]. Increased endothelial cell surface expression of adhesion molecules fosters the initiation of early atherosclerotic processes [19]. We found that chemerin induced a significant increase in protein levels of endothelial cell adhesion molecules. We also demonstrated an increase in secreted endothelial cell adhesion molecules in response to chemerin treatment. The circulating levels of these soluble receptors are considered as potential biomarkers predictive of the severity of cardiovascular disorders [32]. Circulating adhesion molecules have never been directly correlated with chemerin in clinical studies, thus our findings lay the platform for this to be performed.

Initiation, maintenance and progression of atherosclerotic changes are predominantly influenced by surface expression of cell adhesion molecules that have critical effects on inflammation and on the integrity of the vascular wall [33]. Interactions between circulating monocytes and endothelial cells lead to the initiation of early atherosclerotic changes [34]. Extending our findings to a functional monocyte-endothelial adhesion assay, we observed that endothelial cells treated with chemerin resulted in a significant increase of adhered monocytes. Our findings agree with previous reports that have reported chemerin induced adhesion with macrophages [34].

Endothelial recruitment and monocytes adhesion are the earliest detectable events in the pathogenesis of atherosclerosis. The initial steps of endothelium/leucocyte interactions are considered to involve selectins (E-, P-, L- selectin) and I-CAM, V-CAM are crucial for adhesion and migration of leucocytes into the sub-endothelium. So, the elevation of cell adhesive molecules is related to higher morbidity and these cytokines are considered as reliable biomarkers of endothelial activation and inflammation in diabetes and obesity [35]. These dynamic relationships between chemerin and atherosclerosis may give meaningful contribution to the understanding of atherosclerosis pathogenesis in these states leading to early detection and potentially the development of novel therapies [36]. In summary, our novel *in vitro* findings demonstrate a direct mechanism linking chemerin-induced NF-κB activation to functional changes in the endothelium. A limitation of this study is the lack of clinical evidence and *in vivo* animal data. Robust *in vivo* testing must be performed to fully understand the inflammatory properties of chemerin in ECs. Our data provide novel insights into chemerin vascular biology pertinent to atherosclerosis.

## MATERIALS AND METHODS

### Biochemicals and reagents

Full length recombinant human chemerin (2324-CM-025) was obtained from R & D systems, Abingdon, UK; IL-1β peptide: Abcam, Cambridge, UK. BAY 11-7085: Sigma-Aldrich, Gillingham, UK; U0126 (Promega, Southampton, UK); SB203580 (Merck Chemicals Ltd., Nottingham, UK); LY294002 (Merck Millipore, Watford, UK). E-selectin (sc-6940), ICAM-1 (sc-1511) and VCAM-1 (sc-1504) antibodies: Santa Cruz Biotechnology, Inc., Heidelberg, Germany; β-actin antibody: Cell Signaling Technology, Danvers, USA.

### Cell culture

HMEC-1 cells (Centre for Disease Control, Atlanta, USA) were cultured in MCDB media (Sigma-Aldrich, Gillingham, UK) supplemented with 10% Fetal Calf Serum (FCS) (Sigma-Aldrich, Gillingham, UK), 100IU/ml penicillin (Sigma-Aldrich, Gillingham, UK), 100μg/ml streptomycin (Sigma-Aldrich, Gillingham, UK), 5ml of 200mM L-glutamine/500ml of media, 2μM hydrocortisone (Sigma-Aldrich, Gillingham, UK) – hydrocortisone works synergistically with epidermal growth factor to maintain normal colony morphology and growth of HMEC −1 cells in culture [16], 2ng/ml epidermal growth factor (Life Technologies Ltd., Paisley, UK) at 37°C in 5% CO_2_/95% air. Prior to each experiment, cells were serum starved with 1% FCS overnight.

THP-1 cells (LGC, Teddington, UK) were cultured in RPMI 1640 media (Life Technologies Ltd., Paisley, UK) supplemented with 10% heat-inactivated FCS (Sigma-Aldrich, Gillingham, UK), 100 IU/ml penicillin (Sigma-Aldrich, Gillingham, UK), 100μg/ml streptomycin (Sigma-Aldrich, Gillingham, UK) and 2mM L-glutamine (Sigma-Aldrich, Gillingham, UK) and 10mM HEPES (Life Technologies Ltd., Paisley, UK) at 37°C in 5% CO_2_/95% air.

### Transfection of HMEC-1 cells with NF-κB-Luc plasmid

HMEC-1 cells at 80-90% confluence were transfected with pcDNA3.1-NF-κB-Luc (Stratagene, LA Jolla, USA) using Lipofectamine reagent (Life Technologies Ltd., Paisley, UK). After allowing 24 hours of transfection time, cell media were replaced with MCDB 131 media media (Sigma-Aldrich, Gillingham, UK) containing 10% FCS media (Sigma-Aldrich, Gillingham, UK) at a density of 3 × 10^5^ per well in a 6-well plate. Cell lysates were collected, and luminescence was measured using a dual luciferase reporter assay system (Promega, Southampton, UK). For measuring transfection efficiency, a control plasmid with a luciferase gene insert was also transfected and luciferase activity was measured.

### Total RNA extraction and cDNA synthesis

Total RNA was extracted from HMEC-1 cells Qiagen RNeasy Mini Kit according to the manufacturer's guidelines (Qiagen, Crawley, UK). The purity of the extracted RNA was measured by a NanoDrop spectrophotometer. A set concentration of RNA was reverse transcribed into cDNA, by using Moloney murine leukemia virus reverse transcriptase (Fermentas, York, U.K.) and random hexamers (Promega, Southampton, U.K.) as primers, as previously described [17].

### Reverse transcription polymerase chain reaction (RT-PCR)

Real-time quantitative PCR was performed as previously described [16]. Protocol conditions consisted of denaturation at 94°C for 1 minute, then 38 cycles of 94°C for 30 seconds, 60°C for 45 seconds, and 72°C for 30 seconds, followed by extension at 72°C for 7 minutes.

For analysis, quantitative amounts of genes of interest were standardised against the housekeeping gene GAPDH. The RNA levels were expressed as a ratio, using “Delta-delta method” for comparing relative expression results between treatments in real-time PCR [18]. The sequences of the sense and anti-sense primers used were:

E-selectin (198bp) 5′-CAGCCCCCGAAGGGTTT GGTG-3′ and 5′-TCCCGGAACTGCCAGGCTTGA-3′; VCAM-1 (189bp) β-actin (216bp) 5′-AGGTGACG AATGAGGGGACCACA-3′ and 5′-TCGGCTTCCC AGCCTCCAGA-3′; ICAM-1 (189bp) β-actin (126bp) 5′-GAGCTGAAGCGGCCAGCGAG-3′ and 5′-AAGGGGCGGTGCTGCTTTCC-3′; GAPDH (185bp) β-actin (126bp) 5′-GAGTCAACGGATTTGGTCGT-3′ and 5′-GACAAGCTTCCCGTTCTCAG-3′.

10μl of the reaction mixture(s) were subsequently electrophoresed on a 1% agarose gel and visualised by ethidium bromide, using a 1kb DNA ladder (Life Technologies Ltd., Paisley, UK) in order to estimate the band sizes. As a negative control for all the reactions, preparations lacking RNA or reverse transcriptase were used in place of the cDNA. RNAs was assayed from three independent biological replicates.

### Sequence analysis

The PCR products from the cells were purified from the 1% agarose gel using the QIAquick Gel Extraction Kit (Qiagen, Crawley, UK). PCR products were then sequenced in an automated DNA sequencer, and the sequence data were analysed using Blast Nucleic Acid Database Searches from the National Centre for Biotechnology Information, confirming the identity of our products.

### Western blotting

Cells grown in 12-well plates following various treatments were washed with ice cold phosphate buffered saline (PBS) solution and lysed in radio-immunoprecipitation assay (RIPA) buffer (Merck Millipore, Watford, UK).

For experiments involving measurement of secreted proteins, the conditioned media (CM) was collected in sterile Eppendorf tubes, and spun down to remove the dead cells. Protein content in cell lysates and CM were quantified using Bicinchoninic Acid (BCA) assay kit (Sigma-Aldrich, Gillingham, UK). Protein lysates were prepared by mixing cell lysates/CM and SDS-sample buffer (5M urea, 0.17M SDS, 0.4M dithiothreitol and 50mM Tris-HCl, pH 8.0), sonicated, boiled, centrifuged (5000rpm for 2 minutes) and then stored at −80°C until use. 40μg of each sample were subjected to SDS-polyacrylamide gel electrophoresis (10% resolving gel) and transferred to polyvinylidene difluoride (PVDF) membranes (Merck Millipore, Watford, UK). PVDF membranes were blocked in tris buffered saline (TBS) containing 0.1% Tween-20 and 5% bovine serum albumin (BSA) (Sigma-Aldrich, Gillingham, UK) for one hour at room temperature.

The PVDF membranes were then incubated with monoclonal primary mouse-anti-human antibodies for E-selectin, ICAM-1 and VCAM-1 (1:1000 dilutions in 0.1% Tween-20 and 5% BSA) overnight at 4°C. The membranes were washed thoroughly for 60 minutes with TBS-0.1% Tween before incubation with the secondary anti-mouse horseradish peroxidase-conjugated immunoglobulin (Dako, Ely, UK) [1:2000] or secondary anti-rabbit horseradish peroxidase-conjugated immunoglobulin (Dako, Ely, UK) [1:2000], respectively, for one hour at room temperature. Antibody complexes were visualized using chemiluminescence (ECL+GE Healthcare, Little Chalfont, UK). For standardization, the same membranes were stripped and reprobed with monoclonal primary rabbit-anti-human antibody for β-actin (1:10000 dilution).

### Data analysis

The densities were measured using a scanning densitometer coupled to scanning software Scion Image™ (Scion Corporation, Frederick, USA). Standard curves were generated to ensure linearity of signal intensity over the range of protein amounts loaded into gel lanes. Comparisons of densitometric signal intensities for ASAA and β-actin were made only within this linearity range.

### Monocyte-endothelial cell adhesion assay

Monocyte cell adhesion was studied using the Vybrant Cell Adhesion Assay Kit (Life Technologies Ltd., Paisley, UK). THP-1 monocytes were labelled with calcein acetoxymethyl ester (2.5μM final working concentration), swirled gently, and incubated at 37°C for 30 minutes. 100μl/well of resultant suspension containing 5 × 10^5^ cells was added to serum starved endothelial cells cultured in 96-well plate at a final cell density of 0.5-2.0 × 10^6^ cells per ml. After incubating at 37°C for 30 minutes, 75% of cell supernatant volume was aspirated out and discarded. Plate wells were washed twice with 200μl of assay buffer to remove non-adherent THP-1 monocytes. After removal of non-adherent cells, 100μl of assay buffer solution was added to each well, and fluorescence per well (excitation filter 485nm, emission filter 530nm) plate reading was measured using a fluorescence plate reader.

### Statistical methods

Data are mean ± SE. Data were analysed by ANOVA using Tukey's test to correct for multiple comparisons; GraphPad Prism 6; GraphPad Software, San Diego, USA. *P* < 0.05 was considered significant.

## Abbreviations

T2DM: Type 2 Diabetes Mellitus
NF-κB: Nuclear factor kappa-light-chain-enhancer of activated B cells
MAPK: Mitogen-activated protein kinase
PI3K/Akt: Phosphatidylinositol-4,5-bisphosphate 3-kinase/Protein kinase B
E-selectin: Endothelial Selectin
VCAM-1: Vascular Cell Adhesion Molecule-1
ICAM-1: Intracellular Adhesion Molecule-1
IL-1β: Interleukin 1beta
RT-PCR: Reverse transcription polymerase chain reaction
PCOS: Polycystic Ovary Syndrome
VSMC: Vascular smooth muscle cells
EC: Endothelial cells
RIPA: Radio-immunoprecipitation assay
CM: Conditioned media
BCA: Bicinchoninic Acid
PVDF: Polyvinylidene difluoride
TBS: Tris buffered saline
BSA: Bovine serum albumin
